# Biopolymer Nanocomposite Materials Based on Poly(L-lactic Acid) and Inorganic Fullerene-like WS_2_ Nanoparticles

**DOI:** 10.3390/polym13172947

**Published:** 2021-08-31

**Authors:** Mohammed Naffakh

**Affiliations:** Escuela Técnica Superior de Ingenieros Industriales, Universidad Politécnica de Madrid (ETSII-UPM), José Gutiérrez Abascal 2, 28006 Madrid, Spain; mohammed.naffakh@upm.es

**Keywords:** TMDC WS_2_, PLLA, nanomaterials, morphology, crystallization

## Abstract

In the current study, inorganic fullerene (IF)-like tungsten disulphide (WS_2_) nanoparticles from layered transition metal dichalcogenides (TMDCs) were introduced into a poly(L-lactic acid) (PLLA) polymer matrix to generate novel bionanocomposite materials through an advantageous melt-processing route. The effectiveness of employing IF-WS_2_ on the morphology and property enhancement of the resulting hybrid nanocomposites was evaluated. The non-isothermal melt–crystallization and melting measurements revealed that the crystallization and melting temperature as well as the crystallinity of PLLA were controlled by the cooling rate and composition. The crystallization behaviour and kinetics were examined by using the Lui model. Moreover, the nucleating effect of IF-WS_2_ was investigated in terms of Gutzow and Dobreva approaches. It was discovered that the incorporation of increasing IF-WS_2_ contents led to a progressive acceleration of the crystallization rate of PLLA. The morphology and kinetic data demonstrate the high performance of these novel nanocomposites for industrial applications.

## 1. Introduction

Biodegradable polymers can be classified as natural or synthetic polymers according to the source. Synthetic biodegradable polymers based on polyesters, poly(amides), poly(phosphoesters), poly(phosphazenes), poly(orthoesters) and polyanhydride have been widely used and have greatly promoted the development of biomedical fields because of their biocompatibility and biodegradability. Novel polyesters with specific properties have found diverse biomedical applications in drug delivery devices, prosthetics, implants and tissue engineering owing to their tailorable designs or modifications [1,2]. In particular, the use of poly(L-lactic acid) (PLLA), copolymers of lactide/glycolide and lactide/caprolactone (PLGA and PCL-PLLA) to target bio-related applications is highly desirable for practical applications, where the biodegradability of PLLA will allow the incorporation of native tissue into the material over time. The correlation between the morphology, interconnectivity, ability to reabsorb, and interfacial bonding can influence the long-term integrity of the developed material and can control the interaction and integration of new tissue. The mechanical properties, crystallinity, degradation rates and biocompatibility can be controlled by adjusting the monomer ratios [3]. These polyesters and their monomers are endogenous to human metabolism. As with polyesters, polymer hydrophilicity is a key factor that influences the degradation rate. In general, the relative degradation rate of a polyester increases with its hydrophilicity. Polyesters contain the ester linkage between monomer units and degrade by hydrolysis. In particular, PGA is hydrophilic in nature, and it rapidly degrades in vitro aqueous solution and in vivo. PLA is characterized by a slower hydrolysis because it is more hydrophobic than PGA. However, to obtain intermediate degradation rates between PGA and PLA, copolymers of PLGA have been developed [3]. Furthermore, high-performance PLLA can be processed by a wide range of processing techniques such as film casting, extrusion, blow moulding, and fibre spinning due to its better thermal processability [4]. However, its commercial viability has historically been limited by high production costs and poor ability to crystallize [5].

Today, tungsten disulphide (WS_2_) and molybdenum disulphide (MoS_2_) are two of the most popular inorganic nanostructures of transition-metal dichalcogenides (TMDCs), which are promising substances as potential building blocks for high technology applications [6]. They are high-band gap semiconductors with well-known structural anisotropy, such as zero-dimensional (0D), one-dimensional (1D) and two-dimensional (2D) structural anisotropies. The first synthesis of WS_2_ and MoS_2_ inorganic nanotubes (INT, 1D) and fullerene-like nanoparticles (IF, 0D) was reported by Tenne in 1992 and 1993, respectively [7,8]. Since then, a number of engineered inorganic fullerene and nanotubes have been developed to mimic structural reinforcement for polymer blends and nanocomposite components, lubrication, catalysis, rechargeable batteries, solar cells, electronics [9,10] and, more recently, for antiballistic applications [11]. 

In particular, a great benefit of WS_2_ (and of other TMDCs) nanostructures over their carbon equivalents is their low toxicity and biocompatibility, enabling their use for environmental [12] and medical applications [13,14]. Multiple literature sources have reviewed the promising tribological, mechanical and barrier properties of TMDC WS_2_ making them an excellent alternative to carbon nanotubes and graphene as additives for the mechanical reinforcement of polymeric matrices [15,16,17,18]. Coupled with these promising properties, TMDC WS_2_ nanoparticles demonstrate great potential for reinforcement of a variety of biopolymers, showing good manufacturability and performance, as well as reduced manufacturing costs of nanoclays, carbon nanotubes etc. [19,20,21,22,23]. In particular, the incorporation of a low concentration of WS_2_ inorganic nanotubes (0.1 wt%) allowed the crystallization of PLLA at a cooling rate of 10 °C/min, and the crystallization temperature (T_c_) increased by up to 17 °C. This value corresponds to the highest value observed for PLLA nanocomposite materials using MWCNT, SCWCNT, C60 and GO among others [24]. However, the addition of WS_2_ nanosheets into the biopolymer matrix slows down the crystallization rate of PLLA due to the inactive nucleating role of the 2D-WS_2_ [25], whereas the degradation rate of PLLA accelerates markedly on the crystalline PLLA/2D-WS_2_ nanocomposites with the presence of 2D-WS_2_, which is likely related to the accelerated release of degradation products [25]. 

The aim of this paper is to demonstrate the potential of the use inorganic fullerene-like WS_2_ nanoparticles to prepare novel PLLA nanocomposites using a simple melt extrusion. The objective is to analyse the effect of IF-WS_2_ on the crystallization and melting behaviour of pure PLLA.

## 2. Experimental Section

### 2.1. Materials and Processing

The poly(L-lactic acid) (PLLA) purchased from Goodfellow Ltd. (Huntingdon, UK) was used as the base polymer in this study. The IF-WS_2_ nanoparticles (NanoLub) were kindly supplied by Nanomaterials from NanoMaterials Ltd. (Yavne, Israel). They exhibit a quasi-spherical shape with an average aspect ratio of 1.4 and a mean diameter of 80 nm [26]. A range of PLLA/IF-WS_2_ nanocomposites containing 0, 0.1, 0.5 and 1.0, 2.0 wt% IF-WS_2_ were prepared via melt compounding using a micro-extruder (Thermo-Haake Minilab system) at 190 °C with a screw speed of 100 rpm.

### 2.2. Measurements

The dispersion morphology of the PLLA/IF-WS_2_ nanocomposites was observed using an ultra-high field-emission scanning microscope (FESEM), JEOL-JSM7600F (Tokyo, Japan), and a transmission electron microscope (TEM), JEOL-JEM 2100 (Tokyo, Japan).

Non-isothermal melt–crystallization of the PLLA/IF-WS_2_ nanocomposites was measured using a TA Instrument Discovery Differential Scanning Calorimeter DSC 25 (Waters Chromatography, Madrid, Spain). Data were evaluated using the TRIOS software (Waters Chromatography, Madrid, Spain). The samples were heated up to 225 °C and held there for 5 min to eliminate small residual nuclei that might act as seed crystals. Then, the sample was cooled to crystallize at selected constant rates *φ* (in the range from 1 to 20 °C/min). Subsequently, the melting of the samples was conducted using a heating cycle of 10 °C/min over the interval of temperatures between 40 and 225 °C. The exothermic and endothermic curves of heat flow as a function of temperature were recorded and investigated. The degree of crystallinity was calculated as the ratio between the crystallization enthalpy (ΔH_c_) and enthalpy of melting for perfect crystals (ΔH^0^_m_= 93 J/g) [27]. All operations were performed under a nitrogen purge of 50 mL/min. Sample weight varied between 2–5 mg.

## 3. Results

### 3.1. Morphology 

**Figure 1:**
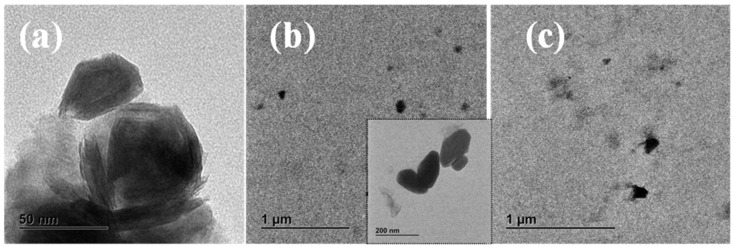
TEM micrographs for (**a**) neat IF-WS_2_ nanoparticles, (**b**) PLLA/IF-WS_2_ (0.1 wt%) and (**c**) PLLA/IF-WS_2_ (1.0 wt%) nanocomposites.

To elucidate the dispersion of IF-WS_2_ in the nanocomposites in detail, Figure 1 illustrates the TEM micrographs of neat IF-WS_2_, PLLA/IF-WS_2_ (0.5 wt%) and PLLA/IF-WS_2_ (1.0 wt%) nanocomposites. Firstly, the IF nanoparticles are closed-cage hollow multi-layered polyhedral nanoparticles with a shape ranging from spheres to ellipsoids. As observed from our previous investigation [26], the particle aspect ratio varies between 1 (spheres) and 2.3, with a mean value of 1.4 and a standard deviation of 0.3. Most visible nanoparticles are quasi-spherical in shape with a diameter in the range of 40–180 nm (mean value of 80 nm). Secondly, all images exhibit highly dispersed IF-WS_2_ nanoparticles with observed dark spots within the PLLA matrix. The TEM analysis reveals that the IF-WS_2_ nanoparticles appear as small aggregates of only a few particles. 

**Figure 2:**
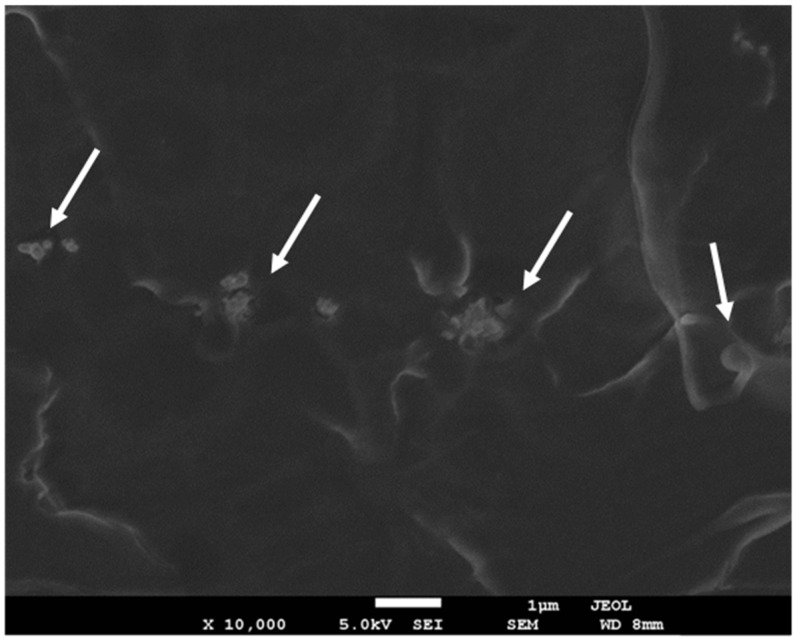
High-resolution SEM micrograph for PLLA/IF-WS_2_ (1.0 wt%) nanocomposites. The white arrows indicate the IF-WS_2_.

Figure 2 shows the morphology of fracture surface of cryogenic-fractured specimens for PLLA/IF-WS_2_ nanocomposites, in which the bright spots are the cross-section of the IF-WS_2_ nanoparticles in the whole examined area. In particular, a uniform distribution of light spots was also observed in all cases of this study (not shown here for brevity), implying that the IF-WS_2_ nanoparticles were well dispersed even without the help of a compatibilizer or modifier, which is in good agreement with the observation made earlier during the TEM assessment.

### 3.2. Non-Isothermal Crystallization and Melting Behaviour

**Figure 3:**
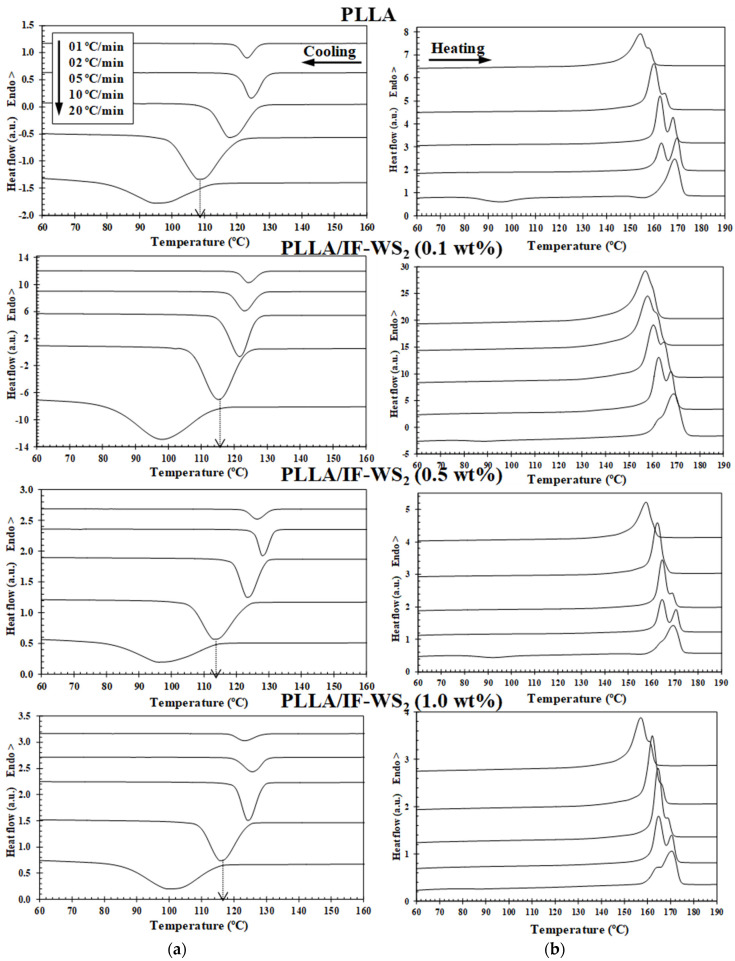
(**a**) Differential scanning calorimetry (DSC) melt–crystallization thermograms of PLLA and PLLA/IF-WS_2_ nanocomposites with nanofiller loadings of 0.1, 0.5 and 1.0 wt% obtained at the indicated cooling rates and (**b**) second-run heating curves obtained at a constant heating rate of 10 °C/min.

It was important to investigate the non-isothermal crystallization to obtain information useful for the industrial applications of PLLA. The DSC cooling and second-run heating curves of pure PLLA and PLLA/IF-WS_2_ nanocomposites are illustrated in Figure 3. The parameters of interest for both crystallization and melting behaviour, that is, melt–crystallization temperature (T_c_), crystallinity (1−λ)_c_, cold-crystallization temperature (T_cc_), cold crystallinity (1−λ)_cc_, melting temperature (T_m_), and melting crystallinity (1−λ)_m_, are summarized in Table 1. For all samples, the crystallization peak became wider and shifted to lower temperatures by increasing the cooling rate. This means that at lower cooling rates, the biopolymer matrix and nanocomposites spent a longer time within the temperature range that promotes sufficient mobility of segments for the growth of crystallization. The addition of IF-WS_2_ nanoparticles to PLLA induces an increase in the crystallization temperature even at the low cooling rate used. Furthermore, if the cooling rate is too high (i.e., 20 °C/min), there will not be enough time for a conformational arrangement allowing for the chains to progress into the crystalline state, and, therefore, they will be amorphous (see the melting curves). Such results indicate that IF-WS_2_ served as a nucleating agent for the crystallization and increased the overall crystallization rate of PLLA. 

**Figure 4:**
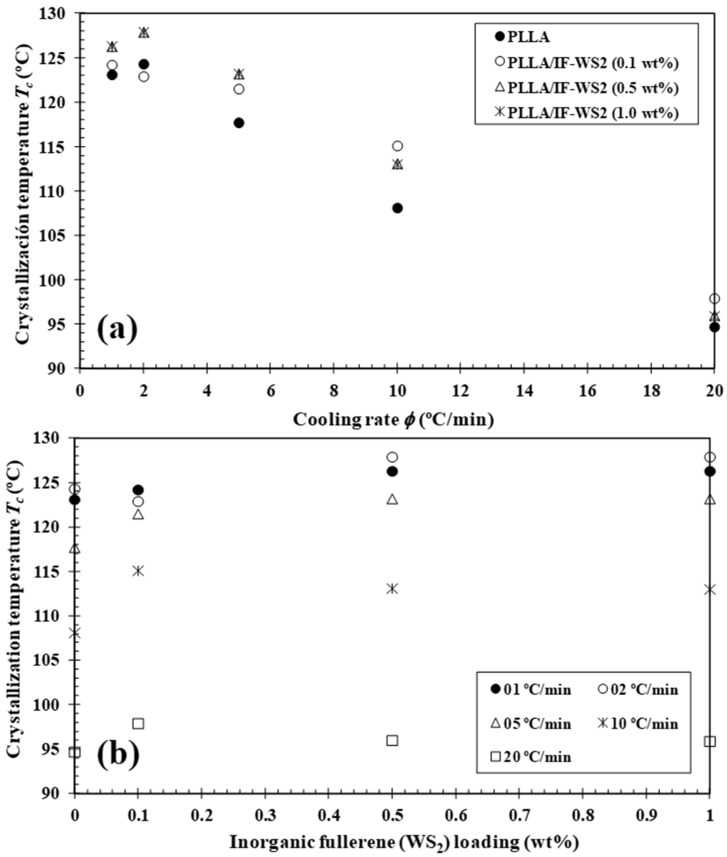
Variation of the melt–crystallization temperature (T_c_) for PLLA/IF-WS_2_ nanocomposites with (**a**) cooling rate and (**b**) composition.

In order to support the previous observations, Figure 4 illustrates the variation in T_c_ with cooling rate and composition, and two clear trends were observed. Firstly, higher cooling rates induced a downward shift in T_c_ to the low-temperature range (Figure 4a). Secondly, the addition of highly compatible IF-WS_2_ with the PLLA matrix may favour the formation of the critical crystal nuclei. This effect was clearly observed to be a function of the composition showing an increase of 7 °C in T_c_, for example, with only 0.1 wt.% IF-WS_2_ at a cooling rate of 10 °C/min (Figure 4b). This highlights the effective nucleating role of IF-WS_2_ in PLLA crystallization, which induces rapid growth of the PLLA crystals on the nanoparticles surface, as suggested in previous literature for PLLA composites reinforced by 1D-WS_2_ inorganic nanotubes [24] as well as other fillers [5,28]. In particular, the effect of the size and shape of nanoparticles on the nucleation of polymer/IFs was deeply explored by Enyashin et al. using a mesoscopic model of Van der Waals’ force field [29]. It was found that in the absence of chemical interaction, the size of the nanoparticle is a dominating factor for the adhesion strength, while the number of sulphide layers composing the cage is not critical. In contrast, 2D-WS_2_ nanosheets were recently found to be ineffective in the crystallization process of PLLA [25]. This was due to the PLLA chains not being able to easily adsorb on the WS_2_ nanosheets, hindering crystal growth. Such differences suggest that the structural anisotropy of layered transition-metal dichalcogenides (0D-, 1D- and 2D-TMDC WS_2_) plays a fundamental role in PLLA crystallization. In the same way, the effect of cooling on the crystallinity (1−λ)_c_ of PLLA and its PLLA/IF-WS_2_ nanocomposites is to be expected, because, at lower rates, macromolecules theoretically have more time for crystallization, which results in fewer defects and thus higher crystal formation (Table 1). However, the influence of IF-WS_2_ on the variation of (1−λ)c values of PLLA appears to only be relevant at a high cooling rate.

**Figure 5:**
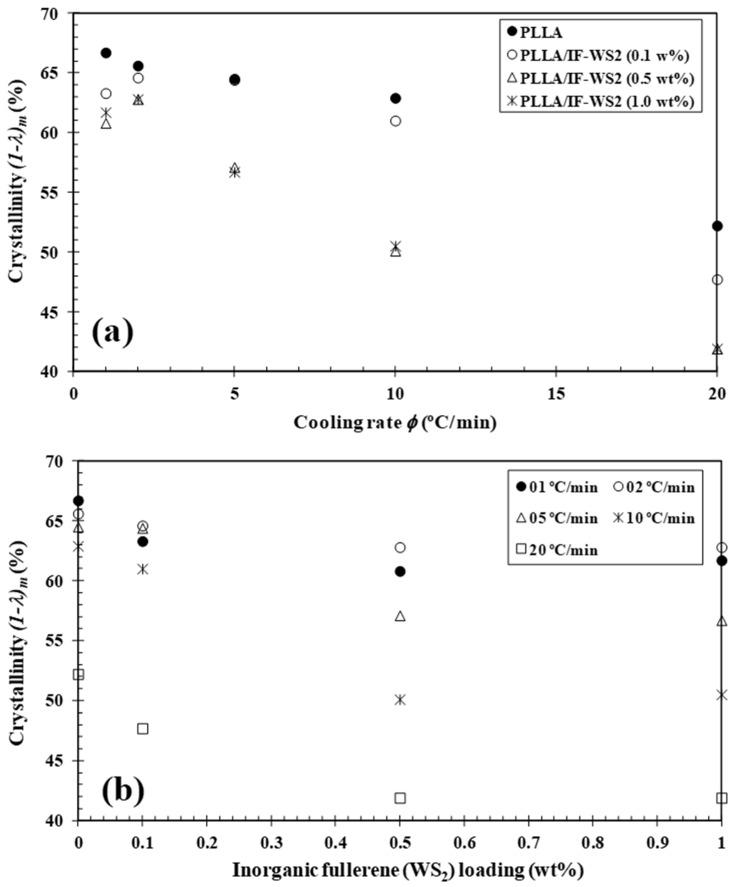
Variation in the melting crystallinity (1−λ)_m_ of PLLA/IF-WS_2_ nanocomposites with (**a**) cooling rate and (**b**) IF-WS_2_ concentration.

The cooling rate also has a significant effect on the melting behaviour of semicrystalline polymers (Figure 3). Two kinds of different melting behaviours could be identified with the cooling rate increasing from 1 to 20 °C/min. As can be seen, the double melting peaks (T_m1_ and T_m2_) appears at around 154 and 157 °C, and the appearance of a double melting endotherm is closely related to IF-WS_2_ concentration and cooling rate. It is necessary to note that the double-melting behaviour of the samples is mainly derived from the melting–recrystallization–remelting processes upon heating [24,30]. During heating, the exothermic peaks related to the cold crystallization process appear for the samples crystallized at higher cooling rates, which suggests that the melt–crystallization process of the samples is incomplete during the cooling. In particular, it was observed that the presence of inorganic fullerene-like WS_2_ nanoparticles induces a decrease in the T_cc_ value of PLLA (e.g., T_cc,PLLA_ = 95.0 °C and T_cc,PLLA/IF-WS2 (0.5 wt%)_ = 88.8 °C), confirming the fact that the addition of IF-WS_2_ enhances the cold crystallization process of PLLA. Figure 5 shows the evolution of the crystallinity (1−λ)_m_ of PLLA/IF-WS_2_ nanocomposites calculated from the double endothermic curves with the cooling rate and composition. In particular, it was noted that the dependence of crystallinity (1−λ)_m_ of PLLA/IF-WS_2_ nanocomposites, shown in Figure 5 both as a function of *φ* and IF-WS_2_ concentration, is consistent with the previously mentioned observation of crystallization temperature curves. This behaviour is expected, because, at slower *φ*, the polymer chains have more time to organize into crystalline domains with fewer defects and, thus, higher (1−λ)_m_. However, the addition of WS_2_ inorganic fullerenes showed a similar trend as a function of *φ*, but a much lower (1−λ)_m_ was observed. These results also confirm that the role of IF-WS_2_ in the variation in the (1−λ)_m_ values of PLLA appears to be only relevant at a high cooling rate.

### 3.3. Lui Analysis

**Figure 6:**
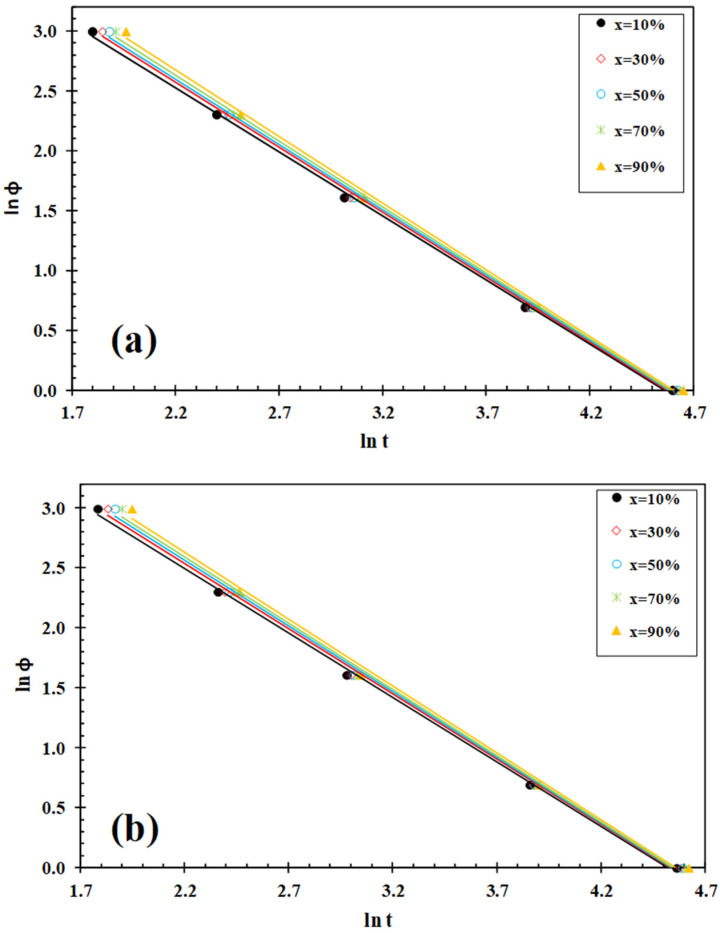
Liu plots for melt–crystallization of (**a**) PLLA and (**b**) PLLA/IF-WS_2_ (0.5 wt%).

The polymer non-isothermal crystallization process can be described by the Lui model [31] by combining the well-known Avrami equation [32] with the Ozawa equation, [33] and it can successfully address the non-isothermal crystallization behaviour of PLLA [5,30]; its final form is given below:

(1) lnφ=lnf(T)−αlnt

where the kinetic parameter *f*(*T*)= [k′(T)/k]^1/m^ refers to the value of the cooling rate that has to be chosen at the unit crystallization time when the measured system amounts to a certain degree of crystallinity; *α* is the ratio of the Avrami exponent n to the Ozawa exponent m (*α* = n/m). According to Equation (1), at a given degree of conversion, the *f*(*T*) parameters of the linear relationship between the plots of ln*φ* vs. ln*t* are obtained (Figure 6). This indicates that the Lui model fits well with the experimental data of the new PLLA/IF-WS_2_ nanocomposites. From the slopes and the intercepts of these lines, the values of *α* and *f*(*T*) could be obtained (Table 2). As can be seen, the values of *f*(*T*) for PLLA and its PLLA/IF-WS_2_ nanocomposites progressively increases with the increase in the relative crystallinity, which indicates that at unit crystallization time, a higher cooling rate should be used to obtain a higher degree of crystallinity. In all cases, the values of *α* are almost constant (i.e., about 1.0–1.1). However, the most relevant observation was the influence of IF-WS_2_ concentration on the value of *f*(*T*) of PLLA for a particular degree of conversion. In particular, it was observed that the value of *f*(*T*) for PLLA is higher than that for the PLLA/IF-WS_2_ nanocomposites, suggesting that the nanocomposites require a lower heating rate to approach an identical degree of crystalline transformation. This implies the acceleration of the crystallization rate of PLLA due to the nucleation effect of IF-WS_2_, while the crystallization mechanism of PLLA remained unchanged in spite of nanoparticle loading. This is because the values of the *α* parameter are approximately the same for both PLLA and the PLLA/IF-WS_2_ nanocomposites (1.0–1.1).

### 3.4. Nucleation Activity

**Figure 7:**
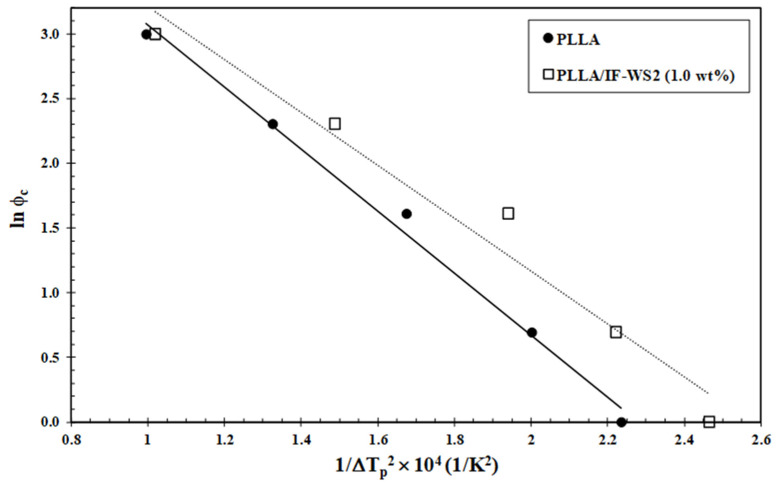
Dobreva plots for evaluating nucleation activity of IF-WS_2_ in PLLA/IF-WS_2_ (1.0 wt%) nanocomposites.

The addition of reinforcing transition-metal dichalcogenides (TMDCs) in polymers can enhance their mechanical properties and thermal stability [10,24,25]. Furthermore, in many cases, substrates such as IF-WS_2_ can act as active or inactive nucleating agents depending on polymer matrix and concentration [34]; therefore, the magnitude of the effect of nucleating activity should be measured. Dobreva and Gutzow [35,36] have developed a simple method to measure the effect of nucleating agents, denominating nucleating activity (*φ*) as follows:

(2) $$\varphi =\frac{B^{*}}{B}$$

where B* stands for the parameter during heterogeneous nucleation, while ***B*** stands for that in homogeneous nucleation. If the foreign substrate is extremely active, *φ* approaches 0, while for inert particles, *φ* approaches 1. ***B*** and B* can both be experimentally determined from the slope of the following equation:

(3) $$\ln\varphi =A-\frac{B\left(\mathrm{or}\;B^{*}\right)}{\Delta T_{p}^{2}}$$

where *φ* is the cooling rate, ***A*** is a constant, and ΔT_c_ denotes the degree of supercooling (T^0^_m_−T_c_). Plots of ln*φ* vs. 1/ΔT_c_ for pure PLLA and PLLA/IF-WS_2_ nanocomposites are shown in Figure 7. It is apparent that a linear relationship is obtained for each sample, assuming the equilibrium melting point T^0^_m_ of PLLA as 195 °C [37]. The values of ***B*** and B* are obtained from the slope of the fitted lines, and the nucleation activity is calculated from their ratio. The corresponding *φ* values are 1.07, 0.80 and 0.81 for PLLA/IF-WS_2_ (0.1 wt%), PLLA/IF-WS_2_ (0.5 wt%) and PLLA/IF-WS_2_ (1.0 wt%), respectively. These results indicate that IF-WS_2_ behaves as an effective nucleating agent when the concentration of IF-WS_2_ is set between 0.5 and 1.0 wt%. However, the excellent nucleation-promoting effect was achieved when the WS_2_ nanoparticles were added as nanotube-like nanoparticles (*φ* = 0.22–0.25) [24]. In contrast, the incorporation of WS_2_ nanosheets induced a significant reduction in the crystallization rate of PLLA due to the inactive nucleating role of WS_2_ [25]. Such differences again suggest that the nanoparticle shape plays a fundamental role in PLLA crystallization.

## 4. Conclusions

In the present work, a series of melt-processable PLLA/inorganic fullerene-like WS_2_ nanoparticles were successfully prepared at various IF-WS_2_ loadings ranging from 0.1 to 1. wt%. TEM and SEM results indicate that IF-WS_2_ is well dispersed in the PLLA matrix without the help of a compatibilizer or modifier. The experimental DSC data show that non-isothermal melt–crystallization peak temperatures are slightly higher in the nanocomposites than in neat PLLA; moreover, the overall non-isothermal melt–crystallization rates are significantly greater in the nanocomposites than in neat PLLA, indicating that IF-WS_2_ acts as a nucleating agent for PLLA. A convenient Lui model appeared to be helpful in elucidating the complex kinetics of PLLA/IF-WS_2_ nanocomposites occurring during continuous cooling. All rate parameters (i.e., *f*(*T*)) suggested that the addition of IF-WS_2_ is an effective approach to speed up the crystallization of PLLA. On the other hand, the study of the nucleation activity using the Gutzow and Dobreva model revealed that the shape of TMDCs nanostructures (0D-, 1D- and 2D-WS_2_) plays a fundamental role in the promotion and/or retardation of PLLA crystallization. In particular, IF-WS_2_ nanoparticles exhibited nucleation activity when the concentration of IF-WS_2_ was set between 0.5 and 1.0 wt%. On subsequent heating, double-melting peaks for PLLA and its nanocomposites can be attributed to a melt–recrystallization mechanism. The addition of IF-WS_2_ appears to have slight influence on the crystallinity value of PLLA, becoming higher as the cooling rate is increased. These results have considerable practical significance for technological processing of PLLA-based materials. PLLA/layered transition metal dichalcogenide (TMDC) nanocomposites can be employed as low-cost biodegradable materials for many eco-friendly and medical implant applications.

## Figures and Tables

**Table 1:** Melt–crystallization and melting parameters of the neat PLLA and PLLA/IF-WS_2_ nanocomposites.

IF-WS_2_ (wt%)	*φ*_c_ (°C/min)	T_c_ (°C)	(1−λ)_c_ (%)	T_cc_ (°C)	(1−λ)_cc_ (%)	T_m1_ (°C)	T_m2_ (°C)	(1−λ)_m_ (%)
0.0	1 2 5 10 20	123.1 124.3 117.7 108.1 94.7	58.1 57.5 53.8 49.7 28.0	- - - - 95.0	- - - - 19.7	154.2 159.0 162.5 163.0 -	157.2 164.8 168.1 169.7 168.7	66.7 65.6 64.5 62.9 52.2
0.1	1 2 5 10 20	124.2 122.9 121.5 115.1 97.9	56.1 57.0 55.8 49.8 33.6	- - - - 88.8	- - - - 1.8	- 157.8 160.3 162.5 163.2	156.8 162.0 164.5 167.5 168.9	63.3 64.6 64.4 61.0 47.7
0.5	1 2 5 10 20	126.3 127.9 123.2 113.1 96.0	55.8 57.0 51.8 43.4 22.5	- - - - 92.1	- - - - 5.7	- - 164.5 164.5 -	157.6 162.5 169.1 170.3 169.2	60.8 62.8 57.1 50.1 41.9
1.0	1 2 5 10 20	126.3 127.9 123.2 113.0 95.9	55.7 57.0 51.8 43.6 23.6	- - - - 92.0	- - - - 5.9	157.6 161.6 164.9 164.5 165.0	160.5 165.9 168.6 170.3 169.2	61.7 62.8 56.7 50.5 41.9

**Table 2:** Values of *α* and *f*(*T*) vs. conversion (x) based on the Liu model for pure PLLA and PLLA/IF-WS_2_ nanocomposites.

IF-WS_2_ (wt%)	*x* (%)	*α*	*f*(*T*)
0.0	10 30 50 70 90	1.07 1.08 1.09 1.10 1.11	4.88 4.96 5.00 5.05 5.12
0.1	10 30 50 70 90	1.06 1.07 1.08 1.09 1.10	4.83 4.90 4.94 4.98 5.04
0.5	10 30 50 70 90	1.07 1.09 1.10 1.10 1.11	4.86 4.92 4.97 5.01 5.08
1.0	10 30 50 70 90	1.06 1.07 1.07 1.08 1.08	4.81 4.86 4.90 4.93 4.98

## Data Availability

The data presented in this study are available on request from the corresponding author.
